# Post-traumatic stress disorder, depression, anxiety, and their predictors among internally displaced persons in a conflict-affected area of Metekel Zone, Northwest Ethiopia: structural equation modeling

**DOI:** 10.3389/fpsyt.2025.1544289

**Published:** 2025-06-13

**Authors:** Solomon Debela Bekeko, Fantahun Ayenew Mekonnen, Rediet Eristu Teklu

**Affiliations:** ^1^ Department of Public Health, Pawi Health Science College, Pawi, Metekel, Ethiopia; ^2^ Department of Epidemiology and Biostatistics, Institute of Public Health College of Medicine and Health Science, University of Gondar, Pawi, Metekel, Ethiopia

**Keywords:** PTSD, depression, anxiety, IDPs, structural equation modeling, Metekel zone

## Abstract

**Introduction:**

Internally displaced persons (IDPs) are people forced to leave their homes due to natural or man-made disasters. Mental health illnesses were linked to conflict and displacement. Post-traumatic stress disorder, depression, and anxiety have the highest rates. However, there are few studies on mental health among internally displaced persons in conflict-affected areas in Ethiopia. To fill this information and methodological gap, structural equation modeling was used to investigate the direct and indirect effects of factors.

**Objective:**

The study aimed to assess the magnitude and determinants of post-traumatic stress disorder, depression, and anxiety among internally displaced persons in the conflict area of Metekel Zone, Northwest Ethiopia, 2023.

**Method:**

A community-based cross-sectional study was conducted among 1,042 participants selected by systematic sampling. Data were collected using face-to-face interviews, and structural equation modeling was used to assess the interrelationship among the variables.

**Result:**

The magnitude of anxiety, post-traumatic stress disorder, and depression was 74.56%, 76.9%, and 79.53%, respectively. Being female, death of a loved one displaced two times, and social support were factors that affected anxiety. The significant factors for post-traumatic stress disorder were being female, death of a loved one, social support, and anxiety. In addition to the significant independent variables, the factors anxiety and post-traumatic stress disorder had an impact on depression.

**Conclusion:**

The magnitude of anxiety, post-traumatic stress disorder, and depression was found to be high. Older-aged IDPs, female IDPs, and those who were not supported by friends or the government were found to be most at risk. Emphasis is needed on the promotion of mental health practices for internally displaced persons due to a conflict, especially for IDPs with poor social support and who have a history of death of loved ones.

## 1 Introduction

The number of internally displaced persons globally stands at over 55 million since 2020, the highest number recorded (1) with the majority of them living in developing nations (2). However, at the end of 2021, there were 59.1 million internally displaced individuals worldwide. From those, 53.2 million were a result of conflict and other forms of violence, and 5.9 million were a result of natural disasters. More than 80% of all internal displacements caused by conflict and violence worldwide in 2021 occurred in Sub-Saharan Africa. The conflict in Ethiopia, the Democratic Republic of the Congo (DRC), Burkina Faso, Somalia, and the Central African Republic (CAR) contributed primarily to the regional total increase of 4.7 million over the previous year (3).

In Ethiopia, conflict and violence led to more than 5.1 million new displacements in 2021, a number that was three times higher than that of 2020 and the biggest annual figure ever recorded for a single nation. Millions of people have been driven from their homes as a result of the conflict that started in November 2020 in Ethiopia’s northern region and grew worse, extending to nearby areas (4).

Post-traumatic stress disorder (PTSD) is a type of mental illness that developed after being exposed to a traumatic or stressful incident. It is characterized by intrusive thoughts, avoidance behavior, mood and cognitive swings, and hyper-arousal that lasts for more than a month after the stressful event (5). Post-traumatic stress disorder, in particular, can have a substantially higher prevalence rate in countries that have recently experienced or are now experiencing conflict (6). The course of PTSD varies in most cases—symptoms appear within 3 months of the traumatic event, while in others, symptoms appear years later (7). PTSD in adults is frequently accompanied by depression, substance addiction, or anxiety disorders (8). Some people recover in 6 months, while others experience symptoms for much longer and the condition becomes chronic in some persons (9). The most common psychological symptoms among internally displaced people and refugees in addition to post-traumatic stress disorder are depression and anxiety (10).

Depression is one of the most common illnesses that affect a person’s emotions, cognition, and behavior. It is characterized by low self-esteem, a depressed mood, hopelessness, helplessness, intense feelings of guilt, sadness, and suicidal and self-harming thoughts (11, 12). Anxiety is a physiological response to a perceived threat, influenced by a person’s beliefs, feelings, and thoughts. It is characterized by worried thoughts, tension, elevated blood pressure, increased heart rate, increased respiration rate, increased pulse rate, sweating, difficulty swallowing, dizziness, and chest pain (13).

Mental illness accounts for approximately 14% of the global burden of disease, with depression and anxiety as leading causes of disability worldwide (14, 15). Notably, developing countries like Ethiopia are impacted by armed conflict, violence, and natural disasters, which can have a substantial impact on mental health and raise the risk of depression, anxiety, and post-traumatic stress disorder in adults (16, 17). Post-traumatic stress disorder, anxiety, and depression are the most common mental health conditions linked to conflict and displacement as previously indicated in the literature (18). As a result of traumatic events during conflicts, post-traumatic stress disorder, depression, and anxiety are recognized as having the greatest prevalence (19).

A meta-analysis indicated that an estimated 242 million adult war survivors living in post-conflict areas have PTSD, and there are an estimated 238 million adult war survivors who have a severe depressive disorder. Around 272.2 million people also suffer from anxiety disorder (20, 21). Another systematic review of 30 studies indicates that PTSD, depression, and anxiety disorders were the most prevalent psychiatric conditions that were looked at in internally displaced persons (IDPs). The study conducted in Nepal showed that the magnitude of anxiety was 80% (22) and in Afghanistan 84.6% (23). The magnitude of PTSD is disproportionately consistently high, ranging from 2.2% to 88.3%; for depression and anxiety disorders, specific estimates range from 5.1% to 81% for depression and from 1% to 90% for anxiety disorders, respectively (24).

During the conflict in the Metekel Zone, thousands of people were displaced, and 107 healthcare institutions were damaged. According to the regional government’s report, the Metekel Zone had the highest number of displaced persons in the region, with 315,000 IDPs, followed by Kamashi and Assosa Zones with 790,00 and 66,000, respectively (25). Internally displaced persons often face disadvantages compared to refugees because they cannot receive assistance from international organizations unless the government requests such assistance (26, 27).

According to the World Health Organization (WHO), depression is a leading cause of disability and significantly contributes to the overall global burden of the disease (28). A systematic review and a meta-analysis were done to show the prevalence rate of depression globally; one in four displaced individuals suffer from depression or depressive symptoms (29).

The mental health of displaced persons should attract research attention for interventions to be designed. It is essential to identify the mental health problems in low-income nations like Ethiopia where conflict and results of forced displacement are very high. However, there is little information available on the magnitude and contributing factors of mental illnesses among internally displaced persons in Ethiopia.

PTSD, depression, and anxiety can be operationalized based on various works of literature. Several studies have identified the risk factors for depression, PTSD, and anxiety in displaced people, such as the lack of social support, unemployment, low educational attainment, the presence of medical conditions, female sex, and widowed or separated marital status, as moderating and risk factors for having a common psychiatric disorder (30–35).

A meta-analysis conducted among North Korean refugees showed that post-traumatic stress disorder, depression, and anxiety symptoms are frequently present in refugees, and the study examined the relationship between PTSD and depression and PTSD and anxiety in North Korean refugees. It was hypothesized that many studies revealed strong associations between PTSD and depression as well as PTSD and anxiety and also that there would be a stronger relationship between PTSD and depression than PTSD and anxiety. PTSD and depression were found to have a significant positive relationship, and also PTSD and anxiety were found to have a significant positive relationship (36).

Studies on displaced populations indicate that the death of an important person was found to be associated with the development of PTSD, depression, and anxiety (37, 38). Another study found that the loss of a family member was one of the risk factors for PTSD in IDPs (39). A study in Ethiopia among IDPs in the post-conflict areas suggests that factors such as the death of a loved one and social support were significantly associated with mental health conditions, depression, and anxiety (38, 40, 41). A study indicated that low social support significantly predicts mental health symptoms like PTSD, depression, and anxiety (29).

Even if little is known about the magnitude and determinants of most common mental illnesses in the conflict setting, these were investigated regularly, as psychological trauma is expected to be widespread among this population (42). Previous studies conducted were without the simultaneous relationship between PTSD, anxiety, and depression; however, in this study, it would be preferable to see them as one possible psychological reaction connected to and coexisting with other responses such as anxiety and depression.

There is scarce evidence about the direct and indirect effects of mental health conditions such as post-traumatic stress disorder, depression, and anxiety among internally displaced persons in conflict areas in Ethiopia. Moreover, studies on mental health problems (PTSD, depression, and anxiety) among internally displaced people affected by conflict were examined using univariate analysis only, whereas more advanced models take into account the complex relationships among mental health problems and their risk factors. The interrelationships between mental illness and risk factors should be considered in the statistical analysis, and both direct and indirect paths should be investigated.

In response to the identified gaps, the structural equation model (SEM) was employed for the simultaneous analyses of the relationships among post-traumatic stress disorder, depression, and anxiety as well as the direct and indirect effect of factors such as socio-demographic and displacement-related, medically confirmed illness, relationship-related affecting post-traumatic stress disorder, depression, and anxiety. Therefore, the objective of this study was to assess the magnitude and determinants of post-traumatic stress disorder, depression, and anxiety among internally displaced persons in the conflict-affected area of Metekel Zone, Northwest Ethiopia, 2023, using structural equation modeling. The findings of this study will also help health professionals, non-governmental organizations (NGOs), and psychological centers develop appropriate plans and interventions to provide evidence-based care for patients with PTSD, depression, and anxiety. Additionally, this study can also serve as baseline data for those who wish to conduct studies in the Metekel Zone, Northwest Ethiopia.

## 2 Method and materials

### 2.1 Study area

This study was conducted in a conflict-affected area of IDP camps in the Metekel Zone, which is in Benishangul Gumuz Regional State Northwest Ethiopia. Metekel Zone is one of the three Benishangul Gumuz regional state administrative zones, with a distance of 546 km from Addis Ababa and 338 km from the regional town of Assosa. Gilgel Beles town is the zone’s administrative center, and it is bounded on the south and southwest by Kamashi, on the west by Sudan, and on the north and east by the Amhara region.

According to the Central Statistical Agency of Ethiopia’s (CSA) 2007 census, this zone has a total population of 276,367, of which 139,119 are men and 137,248 are women. The zone has seven woredas (Dangur, Mandura, Debate, Bullen, Wombera, Guba, and Pawi). The armed conflict known as “war” broke out in the Metekel Zone in 2019 and was centered largely in Dangur, Mandura, Debate, and Bullen woredas.

As a result of the conflict in the Metekel Zone, many people were displaced from the zone. At this time, even if most of the displaced persons returned and lived in post-conflict community, there are still more than 7,000 people who live in IDP camps. There are four recognized IDP sites in Metekel Zone, namely, Dangur, Mandura, Debate, and Bullen sites—with 5,080, 809, 1,008, and 552 IDPs, respectively, and the total of internally displaced persons still living in IDP sites is 7,449.

### 2.2 Study design and period

A community-based cross-sectional study was conducted in the Metekel Zone conflict-affected area in IDP camps among internally displaced persons from March 28, 2023 to April 28, 2023.

### 2.3 Population of the study

#### 2.3.1 Source population

The source population for this study was internally displaced persons who lived in the conflict-affected area of IDP camps in Metekel Zone, Northwest Ethiopia.

#### 2.3.2 Study population

This study included the selected individuals who were internally displaced and lived in IDP camps in the Metekel Zone conflict-affected area.

### 2.4 Inclusion and exclusion criteria

#### 2.4.1 Inclusion criteria

The study included all internally displaced persons who were aged 18 or above and lived in the four IDP sites of the Metekel Zone.

#### 2.4.2 Exclusion criteria

The study excluded IDPs who were not available during the data collection period.

### 2.5 Model specification

To serve the researcher’s interests, each relationship and parameter in the model has to be specified. This step is the most important step that contracts the path diagram, which is the researcher’s hypothesis about the relationship between variables (43). In this study, the hypothesized model for PTSD, anxiety, and depression is supported by theoretical cooperation among IDPs in conflict settings.

After experiencing trauma, people may experience depression, particularly during displacement, whether they physically experience them or only hear about them (44). According to scholars, the most important risk factor for people developing PTSD symptoms is prior trauma, and the more trauma they have experienced, the greater their chances of developing PTSD (45).

### 2.6 Sample size determination and sampling procedures

#### 2.6.1 Sample size determination

For structural equation modeling, the sample size calculation depends on the model complexity. The general rule-of-thumb approach for sample size calculation in structural equation modeling is the ratio of the number of cases (*N*) to the number of model parameters that need statistical estimation (*q*), which might range from five to 20 times per free parameter to be estimated in the hypothesized model (46).

**Figure 1:**
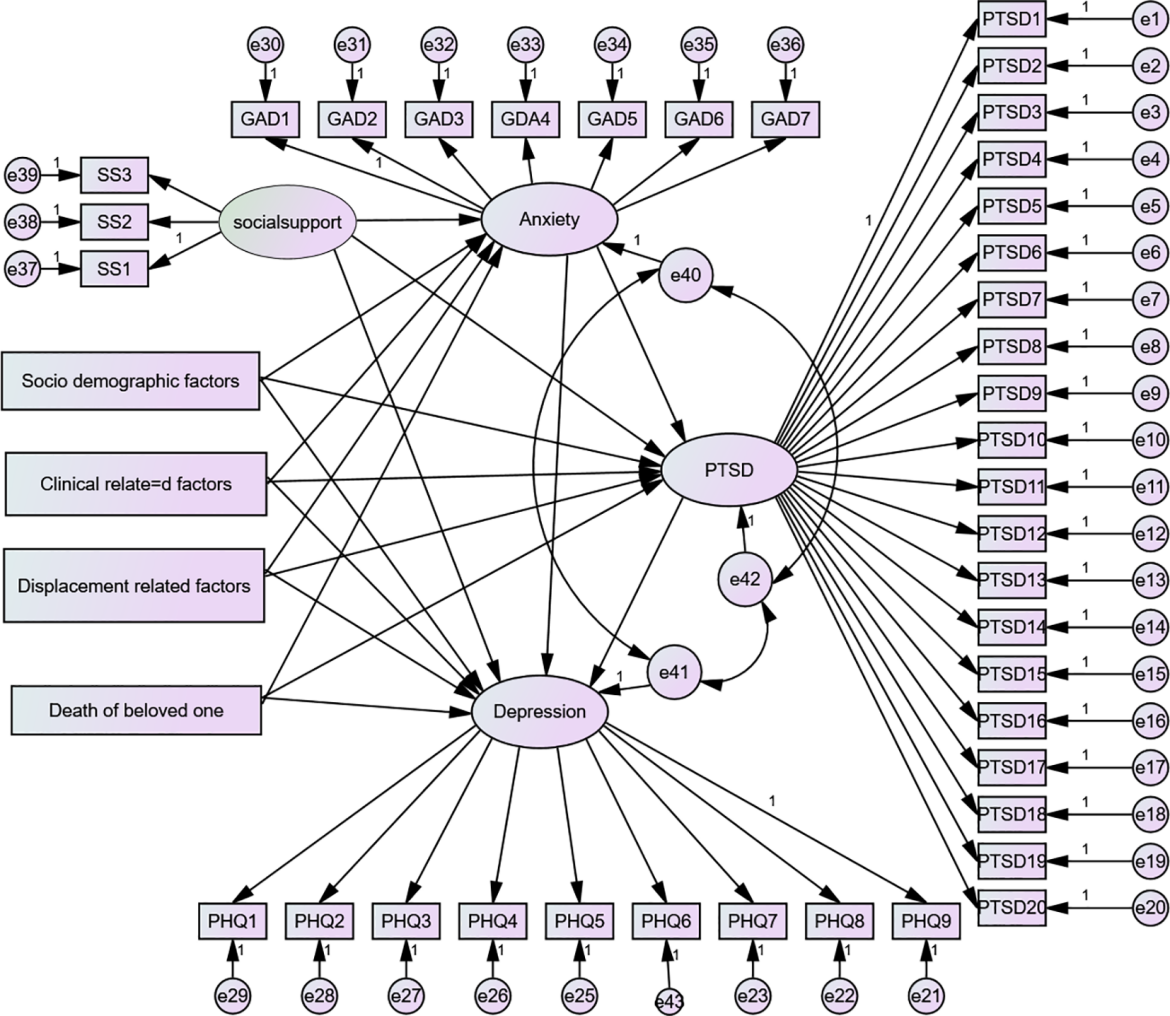
Hypothetical model for PTSD and depression anxiety and determinants among IDPs in the Metekel conflict area, 2023. Rectangles indicate observed variables; circles or ellipses indicate latent (unobserved) variables, including error terms; a single arrow indicates hypothesized directional causal effects or direct effects; on endogenous variables, a line with a single arrowhead indicates the covariance between latent variables, and a double arrow indicates the covariance between latent variables. PTSD1-20, items measuring post-traumatic stress disorder; GDA1-7, items measuring generalized anxiety disorder; PHQ1-9, items measuring depression; SS1–SS3, items measuring social support. Accordingly, from the total of 1,042 recruited individuals, 1,026 were interviewed and completed the questionnaire, and the response rate was 98.5%.

For this study, a ratio of 8:1 was used to obtain the minimum adequate sample size and address the objective. This means that for one free parameter in the hypothesized model, there should be eight respondents. Based on the hypnotized model (**Figure 1**), there are a total of 124 free parameters to be estimated (11 variances for exogenous variables, 33 regression coefficients, which are the coefficients between exogenous observed variables and latent variables, three regression coefficients between the latent variables, three covariances between the errors of latent variables, 35 loadings between latent variables and indicators except for those path coefficients fixed to 1, and 39 error variances for indicator variables). From the thumb rule, it can be used at a ratio of 8:1, and the minimum sample size required is 992 (8 * 124). After adjusting for a 5% non-response rate, the total sample size for this study becomes 1,042 (**Figure 1**).

#### 2.6.2 Sampling procedure

To select the sampling unit from the four IDPs, a systematic random sampling technique was used. A sampling frame was created from the line list of all IDPs that was obtained from the Metekel Zone Disaster and Risk Management Office. We excluded the IDPs who were less than 18 years of age. Then, by arranging the adult IDPs from 1 to *N* lists, the final sampling frame created for the study contained 5,016 from the total of 7,449 IDPs. The sampling interval (*K*) was calculated by dividing the sampling frame by the study sample size, and it was 4.81 ≈ 5. We chose the first IDP from the sampling frame, and we continued choosing respondents based on the sampling interval, or the additional participants were chosen at every *K*-th (5th) interval until the necessary sample size was reached.

### 2.7 Study variables

#### 2.7.1 Dependent variables

The dependent variables include PTSD, depression, and anxiety.

#### 2.7.2 Independent variables

The independent variables are age, sex, marital status, educational status, occupational status, medically confirmed chronic illness, family history of mental illness, frequency of displacement, duration of displacement, death of a beloved one, and social support.

### 2.8 Operational definition and concept definition

#### 2.8.1 Operational definition

Post-traumatic stress disorder: The study participants were classified as having symptoms of PTSD based on the PTSD Checklist for DSM-5 (PCL-5), which consists of 20 items, if their total score is 33 or higher (47).

Depression: The study participants were said to have depression symptoms based on the Patient Health Questionnaire—Nine (PHQ-9) with a score of 10 or higher. Regarding severity measure, the sum of scores of 0–4 indicates no depression, 5–9 indicates mild depression, 10–14 indicates moderate depression, 15–19 indicates moderately severe depression, and 20–27 indicates severe depression (48).

Anxiety: The participants in the study were classified as having symptoms of anxiety based on the Generalized Anxiety Disorder—Seven (GAS-7) if they have a total score of 10 or above. Regarding severity measures, the sum of scores of 4 or less indicates an absence of anxiety, 5–9 mild symptoms, 10–14 moderate symptoms, and 15 or more as severe symptoms of anxiety (49).

Social support: Based on the Oslo 3-item Social Support Scale (OSS-3), the study participants were categorized as having poor social support (3–8 score), moderate social support (9–11 score), and strong social support (12–14) (50).

#### 2.8.2 Concept definition

- Latent variable: It is a variable that cannot be measured directly or observed directly.
- Endogenous variable: A variable is endogenous if any path points to it.
- Exogenous variable: A variable is exogenous if the path point only originates from it or is directed away from it.
- Observed endogenous variable: A variable with a path directed to it that can be measured directly.
- Observed exogenous variable: It is a variable with a path point directed away from it and can be measured directly.
- Indicator variable: It is a variable used to measure a latent or unobserved variable, and it is usually endogenous.
- Latent exogenous: A variable that is not observed is treated as exogenous in our model.

### 2.9 Data collection instruments and procedure

#### 2.9.1 Instruments and data collection tools

Post-traumatic stress disorder was assessed using the PTSD Checklist (PCL-5). PCL-5 is a standardized tool for PTSD that consists of 20 items that correspond to the main symptoms of PTSD, and the scores from each of the 20 items were added up to provide an overall symptom score range from 0 to 80 on the Likert scale of 5 points (0 = not at all, 1 = slightly, 2 = moderately, 3 = quite a little, and 4 = extremely), with a cutoff point of 33 or higher (47). The validity and reliability of PCL-5 have been tested and proven on displaced people and refugees in different countries, for example, in Iraq (Cronbach’s alpha = 0.85) and Zimbabwe (Cronbach’s alpha = 0.92) (51, 52). This tool was used in studies conducted in Ethiopia, in Debre Berhan among IDPs (Cronbach’s coefficient was 0.878), and in North Shoa Zone among conflict-affected people (Cronbach’s coefficient was 0.90), showing an excellent internal consistency of the items (41, 53).

Depression was measured using the Patient Health Questionnaire 9 (PHQ–9), which was used to evaluate depression. With its unique focus on the nine diagnostic criteria for DSM-IV depressive disorders, the PHQ-9 is a commonly utilized instrument in primary healthcare settings. The Patient Health Questionnaire—Nine (PHQ-9) has nine items assessed on a four-point Likert scale with a total score range of 0 to 27, where 0 = “not at all,” 1 = “several days,” 2 = “more than half of the days,” and 3 = “almost every day,” with a cutoff score of 10 or higher being considered depressive. Scores can be transferred into the severity measure, where a sum of scores of 0–4 indicates no depression, 5–9 indicates mild depression, 10–14 indicates moderate depression, 15–19 indicates moderately severe depression, and 20–27 indicates severe depression (48, 54). The PHQ-9 has been validated in Ethiopia and used among refugee-displaced persons, and the reliability coefficient Cronbach’s was above 0.89, showing good internal consistency (55, 56).

Anxiety was assessed using Generalized Anxiety Disorder—Seven (GAD-7), which is a brief measure of anxiety that has seven items and is assessed on a four-point Likert scale with a total score range of 0 to 21, where 0 = “not at all,” 1 = “several days,” 2 = “more than half of the days,” and 3 = “almost every day.” Scores can be transferred into the severity measure, where a sum score of 4 or less is regarded as the absence of anxiety, 5–9 as a mild symptom, 10–14 as a moderate symptom, and 15 or more as a severe symptom of anxiety (49). The GAD-7 has adequate psychometric validity, including convergent validity, internal consistency, and test–retest reliability in various populations (49, 57).

Social support was measured using the Oslo Social Support Scale-3 (OSSS-3), the scores of which range from 3 to 14, and respondents who receive scores 3 to 8 are considered to have poor social support, those who receive scores 9 to 11 are considered to have moderate social support, and those who receive scores 12 to 14 are considered to have strong social support (50).

#### 2.9.2 Data collection procedures

Primary data were collected by an interviewer using a structured questionnaire prepared by the investigator based on previous studies. The questionnaire included sociodemographic factors, clinical-related factors, displacement-related factors, and relationship-related factors. Individual items (indicators) cross-referencing the latent variables were used in the questionnaire, including items measuring post-traumatic stress disorder (20 items), depression (nine items), and anxiety (seven items). To collect the data, the English version of the collected data was translated into Amharic and back to English to ensure consistency.

### 2.10 Data quality control

Data were gathered using the structured questionnaire under the guidance and supervision of experienced and trained data collectors and supervisors. The supervisors received training before data collection to understand the questionnaire. The data collectors also received a 1-day training on the objective of the study, the techniques of data collection, the content of the questionnaire, and the concern of confidentiality. There were four trained BSc public health professionals and two health extension workers who were native in the local languages. The data collectors were supervised by trained BSc psychiatrists from Pawi General Hospital during the data collection process. Any ambiguity was resolved through communication with the supervisors. Furthermore, the principal investigator followed the entire data collection process.

Post-traumatic stress disorder, depression, and anxiety scales have been adopted and used many times in the Ethiopian context in post-conflict-affected areas, among internally displaced persons and refugees (38, 41, 57, 58). Before data collection for understandability, the questionnaires were pre-tested in Assosa Zone Bambasi IDP camp by taking 5% (50) of the total sample size who were not included in the study.

### 2.11 Data processing model building and analysis

#### 2.11.1 Data processing

The collected data were coded and entered into Epi Data Version 4.6 and exported to STATA Version 17 and Amos Version 21 for further analysis. Descriptive analyses were done using texts, tables, graphs, charts, and figures for data summarization, and the normality of the data was maintained by using the bootstrapping method. The reliability of the tools for PTSD, depression, and anxiety was assessed, and Cronbach’s alpha reliability coefficients were used in each construct (PTSD, depression, and anxiety) for the current study. If Cronbach’s alpha coefficients are greater than 0.7, it is considered as satisfactory. Individual items (indicators) cross-referencing the latent variables were used in the questionnaire, including items measuring post-traumatic stress disorder (20 items), depression (nine items), and anxiety (seven items).

### 2.12 Confirmatory factor analysis

Factor analysis can be categorized into two groups: exploratory and confirmatory. In explanatory factor analysis, factors are discovered through relationships between variables. Furthermore, the observed variables may be loaded onto one or more factors. However, in the confirmatory factor analysis, the existing data support the theoretically predicted factor structure or predetermined factor that can be loaded on an observed variable in the confirmatory factor analysis. Explanatory factor analysis is used to extract the latent variables from the observable data. However, the confirmatory factor analysis uses the gathered data to confirm previously discovered scales (59).

The CFA model was used to start the statistical analysis because there is enough prior knowledge for this study to imply that some latent variables or factors can represent the instruments that are specifically designed to have items referring to a particular latent variable, such as the PTSD, depression, and anxiety instruments. Hence, the statistical analysis can be started with the CFA model.

Adequate sample size: The sample size to be considered in the structural equation model is large. A “sufficient” sample size is necessary for the structural equation model to provide credible results. The sample size has an effect on various fit indices in structural equation modeling (60). In this study, the value of Kaiser–Meyer–Olkin (KMO) used for test sampling adequacy was above 0.7 for the particular constructs, and the overall was 0.89. Bartlett’s test of sphericity results for all constructs was significant (*p* < 0.05). Therefore, the sample is adequate, and factor analysis (FA) was evidenced (**Table 1**).

**Table 1:** Kaiser–Meyer–Olkin (KMO) and Bartlett’s test of sphericity results of latent variables used for test sampling adequacy, Metekel Zone, 2023.

Factors	KMO	Bartlett’s test of sphericity	
		Chi-square	*p*-value
PTSD	0.808	2,530.5	0.000
Anxiety	0.706	1,116.6	0.000
Depression	0.702	1,252.0	0.000
Overall	0.892	8,484.3	0.000

**Table 2:** Several fit indexes and their cutoff criteria.

Fit indices	Good fit values	Acceptable fit values
CMIN/DF	0 < CMIN/DF < 2	1 < CMIN/DF < 5
Comparative Fit Index (CFI)	≥0.95	0.95 ≤ CFI ≤ 1
TLI Tucker Lewis Index (TLI)	≥0.95	0.90 ≤ TLI ≤ 1
Adjusted Goodness of Fit Index (AGFI)	≥0.95	0.90 ≤ GFI ≤1
Normed Fit Index (NFI)	≥0.95	0.90 ≤ NFI ≤ 1
RMSEA	0 < RMSEA < 0.05	0.05 < RMSEA < 0.08

Multi-collinearity: In the structural equation model, it is assumed that there is no relationship between the independent variables. It occurs when there is a high degree of correlation between two or more explanatory factors. This is a concern since it can be challenging to determine which of them best accounts for any common variance in the result. Additionally, it implies that the two variables can represent the same factor. Variance inflation factor (VIF) and tolerance were used to test multi-collinearity (61). The maximum VIF in this study is 2.86, which indicates the absence of multi-collinearity.

### 2.13 Ethical consideration

Ethical approval was obtained from the Institutional Review Board (IRB) of University of Gondar, College of Medicine and Health Science, Institute of Public Health. The ethical approval letter was submitted to the Metekel Zone disaster and risk management office for those who are concerned about IDPs. After getting permission and a supportive letter from the zone, written consent was taken from the participants. Then, data collection was started after explaining the aim of the study, confidentiality, and its possible benefits to the participants.

## 3 Results

### 3.1 Socio-demographic characteristics and clinical-, displacement-, and relationship- characteristics of the respondents

A total of 1,026 individuals were interviewed and completed the questionnaire, and the response rate was 98.5%. Approximately 56.43% of the study participants were female. The age of the participants ranged from 20 to 82 years, with a median age of 44 years, and the inter-quartile range (IQR) was 35–55 years. More than two-thirds (68.32%) of the participants were married, and more than half (58.48%) were unable to read and write. The majority (81.77%) of the respondents were farmers. Regarding the duration of displacement, the majority (96.20%) of the displaced individuals stayed in the camp for more than 12 months, and more than half (59.45%) of the respondents were displaced once. The majority (99.03%) of the participants had poor social support, and no one had strong social support (**Table 3**).

**Table 3:** Socio-demographic characteristics and clinical-, displacement-, and relationship-related characteristics of IDP respondents in Metekel Zone, 2023 (*n* = 1,026).

Variable	Category	Frequency (*n* = 1,026)	Percentage (%)
Socio-demographic characteristics			
Age in years	Continuous	Median 44 years and IQR: 35–55 years	
Sex	Male	447	43.57%
	Female	579	56.43%
Marital status	Single	83	8.09%
	Married	701	68.32%
	Divorced	129	12.57%
	Widowed	113	11.01%
Educational status	Unable to read and write	600	58.48%
	Primary	321	31.29%
	Secondary	65	6.34%
	College and above	40	3.90%
Occupational status	Farmer	839	81.77%
	Marchant	92	8.97%
	Employed	33	3.22%
	Unemployed	9	0.88%
	Student	20	1.95%
	Daily labor	33	3.22%
Displacement-related			
Duration of displacement	Less than 12 months	39	3.80%
	Greater than 12 months	987	96.20%
Frequency of displacement	Once	610	59.45%
	Two times	416	40.55%
Clinically related factors			
Family history of mental illness	No	991	96.59%
	Yes	35	3.41%
History of chronic diseases	No	785	76.61%
	Yes	240	23.39%
Relationship-related factors			
Death of beloved one	No	778	75.83%
	Yes	248	24.17%
Social support	Poor	1016	99.03%
	Moderate	10	0.97%
	Strong	–	–

### 3.2 The magnitude of post-traumatic stress disorder, depression, and anxiety

#### 3.2.1 Magnitude of anxiety

**Figure 2:**
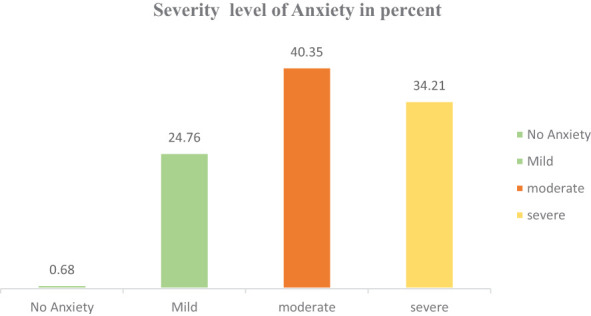
Proportion of anxiety severity level among IDPs in Metekel Zone, 2023.

In this study, the overall magnitude of anxiety was 74.56% (95% CI = 71.78%, 7701%). Regarding the level of severity, 261 (25.44%) of the respondents had mild anxiety, 414 (40.35%) had moderate anxiety, and 351 (34.21%) of the respondents had severe anxiety (**Figure 2**).

#### 3.2.2 Magnitude of post-traumatic stress disorder

**Figure 3:**
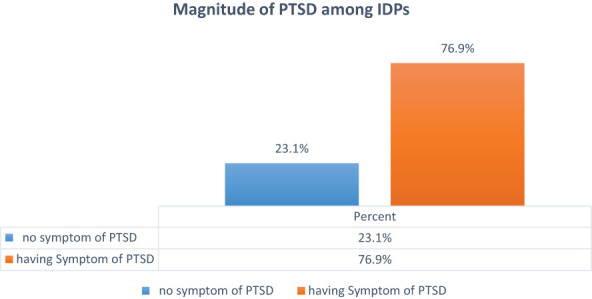
Proportion of post-traumatic stress disorder among IDPs in Metekel Zone, 2023.

Based on the PTSD Checklist for DSM-5 (PCL-5) with a cutoff point of 33 and above, of the total internally displaced persons who participated in this study, more than two-thirds (76.9%, 95% CI: 74.2%, 79.4%) had symptoms consistent with PTSD. Of the total participants, 540 (52.63%), 570 (55.56%), and 720 (70.18%) had reported that they had trouble experiencing positive feelings (being unable to feel happiness), unwanted memories of the stressful experience, and never feeling distant or cut off from other people, respectively (**Figure 3**).

#### 3.2.3 Magnitude of depression

**Figure 4:**
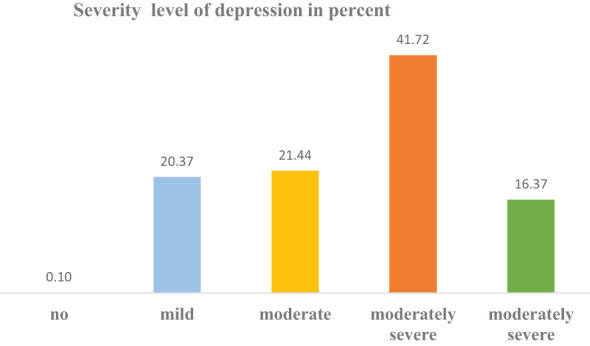
Proportion of depression severity level among IDPs in Metekel Zone, 2023.

In this study, the overall magnitude of depression was 79.53% (95% CI: 76.93%, 81.86%). Regarding the level of severity, 210 (20.37%) of the respondents had mild depression, 220 (21.44%) had moderate depression, 428 (41.72%) had moderately severe depression, and 168 (16.37%) of the respondents had severe depression (**Figure 4**).

### 3.3 Structural model

Structural modeling is a statistical technique that allows a set of relationships among one or more independent variables, either continuous or discrete, and one or more dependent variables, either continuous or discrete, to be examined. Both dependent and independent variables can be either factor or measured variables. In our case, the structural model is recursive with 10 common predictor variables for those outcome variables (anxiety, PTSD, and depression). The model had factors as independent variables: one-factor variable (social support) for anxiety, two-factor variables (social support and anxiety) for PTSD, and three factors variables (social support, anxiety, and PTSD) for depression.

**Figure 5:**
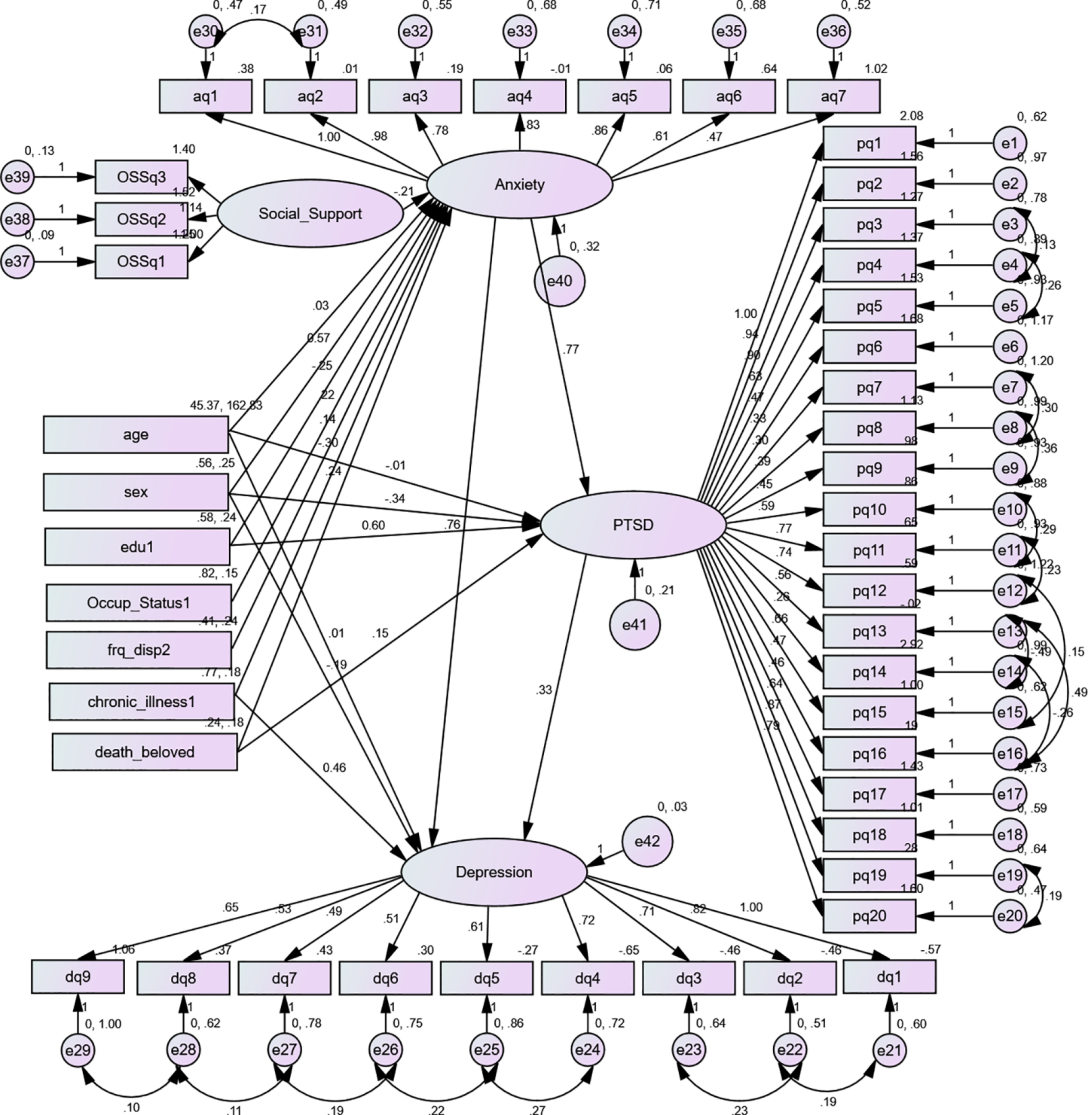
Final structural equation model showing the association between predictors and outcomes and between anxiety, post-traumatic stress disorder, and depression among conflict-affected internally displaced persons in Metekel Zone, 2023. Unstandardized estimates: Rectangles indicate observed variables; circles or ellipses indicate latent (unobserved) variables, including error terms; a single arrowhead indicates directional causal effects, or direct effects, on endogenous variables; a line with a double arrowhead indicates the covariance between latent variables or error terms. pq1-pq20, items measuring post-traumatic stress disorder; aq1–aq7, items measuring generalized anxiety disorder; dq1–dq9, items measuring depression; SS1–SS3, items measuring social support; PTSD, post-traumatic stress disorder; sex, being of female gender; edu1, educational status (unable to read and write or no formal education); Occup_Status1, having occupational status farmer; frq_disp2, frequency of displacement being displaced two times; chronic_illness1, having no medically confirmed chronic illness; death_beloved, having a history of the death of a beloved one during the past 6 months.

Both structural components (relationships between latent or observable variables) and measurement components (relationships between a latent variable and its indicators or items) are included in the final fitted model, as indicated in **Figures 1**–**5** and **Tables 1**–**7**. Only significant predictor variables were used in the final model, which was reasonably straightforward and best fitted. **Figures 1**–**5** displays the final fitted model with significant path coefficients at 0.05 level of significance. The final model includes 39 indicator variables or items, seven observed exogenous variables, socio-demographic characteristics (age, sex, educational status, and occupational status), a displacement-related factor (frequency of displacement), clinically relevant factors (medically confirmed chronic illness), and relationship-related factors (death of a loved one and unobserved exogenous: social support), and two unobserved endogenous variables (anxiety and PTSD) were included in the final model.

The unstandardized regression coefficients showed how much the dependent variables or mediating variables changed as a result of the independent variables or predictors changing values. Error terms were inter-correlated based on the modification indices. The hypothesized relationship between the three latent variables and other exogenous observed variables was examined. Categorical variables were dummy-coded as needed. Non-significant variables and non-significant paths were removed to maintain a minimal model or simplified model.

Variables such as marital status, duration of displacement, and family history of mental illness were excluded from the final model since their regression weight on all latent variables were not statistically significant at the alpha level of 0.05, whereas variables such as age, sex, educational status, occupational status, frequency of displacement, confirmed history of chronic illness, and death of a beloved one were included in the final model because their contribution were statistically significant at least on one of the latent variables in the model (**Figure 5**).

### 3.5 Test of model fitness indices for the final fitted SEM model

In this study, the model fit indices satisfy the hypothetical model according to the recommended acceptable level of the indices (**Table 4**).

**Table 4:** Model fit indices of the final fitted structural equation model.

Fit indices	Results
CMIN/DF	2.980
Adjusted Goodness of Fit I index (AGFI)	0.913
Comparative fit index (CFI)	0.941
Tucker Lewis Index (TLI)	0.921
Incremental Fit Index (IFI)	0.930
Normed Fit Index (NFI)	0.901
Root mean square error of approximation (RMSEA)	0.042
Akaike information criterion (AIC)	1324.7

### 3.6 Factors associated with anxiety among IDPs in Metekel Zone

The independent variables (age, educational status, occupational status, medically confirmed chronic illness, frequency of displacement, and social support) had a statistically significant direct relation with anxiety. Age (adjusted *β* = 0.30, 95% CI: 0.027, 0.033), being female (adjusted *β* = 0.566, 95% CI: 0.554, 0.580), being a farmer (adjusted *β* = 0.220 95% CI: 0.057, 0.291), and being displaced two times (adjusted *β* = 0.136, 95% CI: 0.089, 0.184) had a direct positive effect on anxiety.

On the other hand, having a history of death of a beloved one (adjusted *β* = 0.241, 95% CI: 0.192, 0.294), being unable to read and write (*β* = -0.249, 95% CI: -0.327, -0.176), not having a medically confirmed chronic illness (adjusted *β* = -0.300, 95% CI: -0.389, -0.222), and social support had a direct negative effect (adjusted *β* = -0.210, 95% CI: -0.346, 0.145) on anxiety (**Table 5**).

**Table 5:** Direct, indirect, and total effect of significant socio-demographic, clinical, relationship, and displacement-related factors on anxiety among internally displaced persons in Metekel Zone, 2023 (unstandardized estimate).

Variables	Direct effect Estimate (95% CI)	Indirect effect Estimate (95% CI)	Total effect Estimate (95% CI)
DV: Anxiety			
Age	0.030 (0.027, 0.033)^a^	—	0.030 (0.027, 0.033)^a^
Sex			
Female	0.566 (0.554, 0.580)^a^	—	0.566 (0.554, 0.580)^a^
Male	Ref.	—	Ref.
Educational status (unable to read and write)	-0.249 (-0.327, -0.176)^a^	–	-0.249 (-0.327, -0.176)^a^
Occupational status			
Occupational status (farmer)	0.220 (0.149, 0.291)^a^	—	0.220 (0.149, 0.291)^a^
Medically confirmed chronic illness			
No	-0.300 (-0.389, -0.222)^a^	—	-0.300 (-0.389, -0.222)^a^
Yes	Ref.	—	Ref.
Death of beloved one			
Yes	0.241 (0.192, 0.294)^a^		0.241 (0.192, 0.294)^a^
No	Ref.	—	Ref.
Frequency of displacement			
One time	Ref.	—	Ref.
Two times	0.136 (0.089, 0.184)^a^	—	0.136 (0.089, 0.184)^a^
Social support	-0.210 (-0.346, -0.145)^a^	—	-0.210 (-0.346, -0.145)^a^

DV, dependent variable; Ref., reference category; CI, confidence interval.

a The CI did not include a null value (*β* = 0).

### 3.7 Factors associated with PTSD among IDPs in Metekel Zone

Independent variables such as age, sex, and educational status (unable to read and write) were significantly related to post-traumatic stress disorder both directly and indirectly. Age had a direct negative effect (adjusted *β* = -0.012, 95% CI: -0.015, -0.010) and an indirect positive effect (adjusted *β* = 0.023, 95% CI: 0.019, 0.027), resulting in a total positive effect of 0.010 (adjusted *β* = 0.010, 95% CI: 0.008, 0.013), on PTSD. In addition, being female had a direct negative effect (adjusted *β* = -0.336, 95% CI: -0.393, 0.286) and an indirect positive effect (adjusted *β* = 0.773, 95% CI: 0.701, 0.852), leading total positive effect of 0.436 (adjusted *β* = 0.436, 95% CI: 0.377, 0.496), on PTSD.

Educational status (unable to read and write) had a direct positive effect and an indirect negative effect, resulting in a total positive effect of 0.487, on PTSD (adjusted *β* = 0.487, 95% CI: 0.438, 0.528). Being a farmer and being displaced two times were significantly associated with PTSD indirectly (adjusted *β* = 0.170, 95% CI: 0.114, 0.229 and adjusted *β* = 0.105, 95% CI: 0.069, 0.143, respectively).

Having a history of the death of a beloved one had a direct positive effect (adjusted *β* = 0.154, 95% CI: 0.102, 0.205) and an indirect positive effect (adjusted *β* = 0.187, 95% CI: 0.146, 0.232), resulting in the total positive effect of 0.341 (adjusted *β* = 0.341, 95% CI: 0.289, 0.394), on PTSD. Social support had an indirect negative effect (adjusted *β* = -0.162, 95% CI: -0.222, -0.110) on PTSD. Anxiety was significantly related to PTSD (adjusted *β* = 0.773, 95% CI: 0.701, 0.852) (**Table 6**).

**Table 6:** Direct, indirect, and total effect of significant socio-demographic and clinical-, relationship-, and displacement-related factors and anxiety on PTSD among internally displaced persons in Metekel Zone, 2023 (unstandardized estimate).

Variables	Direct effect Estimate (95% CI)	Indirect effect Estimate (95% CI)	Total effect Estimate (95% CI)
DV: PTSD			
Age	-0.012 (-0.015, -0.010)^a^	0.023 (0.019, 0.027)^a^	0.010 (0.008, 0.013)^a^
Sex			
Female	-0.336 (-0.393, 0.286)^a^	0.773 (0.701, 0.852)^a^	0.436 (0.377, 0.496)
Meal	Ref.	Ref.	Ref.
Educational status (unable to read and write)	0.603 (0.589, 0.618)^a^	-0.116 (-0.167, -0.075)^a^	0.487 (0.438, 0.528)^a^
Occupational status (farmer)	—	0.170 (0.114, 0.229)^a^	0.170 (0.114, 0.229)^a^
Frequency of displacement			
One time	Ref.	Ref.	Ref.
Two times	—	0.105 (0.069, 0.143)^a^	0.105 (0.069, 0.143)^a^
Medically confirmed chronic illness			
Yes	Ref.	Ref.	Ref.
No	—	-0.232 (-0.292, -0.177)^a^	-0.232 (-0.292, -0.177)^a^
Death of beloved one			
Yes	0.154 (0.102, 0.205)^a^	0.187 (0.146, 0.232)^a^	0.341 (0.289, 0.394)^a^
No	Ref.	Ref.	Ref.
Social support	—	-0.162 (-0.222, -0.110)^a^	-0.162 (-0.222, -0.110)^a^
Anxiety	0.773 (0.701, 0.852)^a^	—	0.773 (0.701, 0.852)^a^

DV, dependent variable; Ref., reference category; CI, confidence interval; PTSD, post-traumatic stress disorder.

a The CI did not include a null value (*β* = 0).

### 3.8 Factors associated with depression among IDPs in Metekel Zone

From our study, variables such as age, sex, medically confirmed chronic illness, anxiety, and PTSD were significantly related to depression both directly and indirectly. Age had a direct positive effect (adjusted *β* = 0.008, 95% CI: 0.006, 0.011) and an indirect positive effect (adjusted *β* = 0.026, 95% CI: 0.023, 0.029), leading to a total positive effect of 0.034 (adjusted *β* = 0.034, 95% CI: 0.032, 0.037), on depression, and being female had a positive effect (adjusted *β* = 0.719, 95% CI: 0.665, 0.771) on depression. Having no medically confirmed chronic illness had a direct and an indirect effect on depression, resulting in a total positive effect (adjusted *β* = 0.324, 95% CI: 0.275, 0.364).

In this study, having a history of the death of a beloved one had an indirect positive effect (adjusted *β* = 0.296, 95% CI: 0.248,0.349), being displaced two times had an indirect positive effect (adjusted *β* = 0.138, 95% CI: 0.091, 0.187), being a farmer had an indirect positive effect (adjusted *β* = 0.224, 95% CI:0.153, 0.341), and social support had an indirect negative effect (adjusted *β* = -0.214, 95% CI: -0.285, -0.148) on depression, and all were significantly related to depression indirectly.

Anxiety (directly and indirectly) and PTSD (directly) were significantly related to depression. Anxiety had a significant direct effect (adjusted *β* = 0.762, 95% CI: 0.643, 0.893) and an indirect (*β* = 0.254, 95% CI: 0.185, 0.339) positive effect on depression, resulting in a total positive effect of 1.016 (adjusted *β* = 1.016, 95% CI: 0.952, 1.094). PTSD had a significant direct (adjusted *β* = 0.329, 95% CI: 0.253, 0.407) positive effect on depression in this study (**Table 7**).

**Table 7:** Direct, indirect, and total effect of significant socio-demographic and clinical-, relationship-, and displacement-related factors, anxiety, and PTSD on depression among internally displaced persons in Metekel Zone, 2023 (unstandardized estimate).

Variables	Direct effect Estimate (95% CI)	Indirect effect Estimate (95% CI)	Total effect Estimate (95% CI)
DV: Depression			
Age	0.008 (0.006, 0.011)*	0.026 (0.023, 0.029)*	0.034 (0.032, 0.037)*
Sex	0	0	0
Female	-0.187 (-0.262, -0.126)*	0.906 (0.824, 1.000)*	0.719 (0.665, 0.771)*
Male	Ref.	Ref.	Ref.
Occupational status (farmer)	—	0.224 (0.153, 0.296)*	0.224 (0.153, 0.341)*
Medically confirmed chronic illness			
Yes	0.460 (0.452, 0.483)*	-0.142 (-0.200, -0.099)*	0.324 (0.275,0.364)
No	Ref.	Ref.	Ref.
Death of beloved one			
Yes	—	0.296 (0.248,0.349)*	0.296 (0.248,0.349)*
No	Ref.	Ref.	Ref.
Frequency of displacement			
One time	Ref.	Ref.	Ref.
Two times	—	0.138 (0.091, 0.187)*	0.138 (0.091, 0.187)*
Social support	—	-0.214 (-0.285, -0.148)*	-0.214 (-0.285, -0.148)*
Anxiety	0.762 (0.643, 0.893)*	0.254 (0.185, 0.339)*	1.016 (0.952, 1.094)*
PTSD	0.329 (0.253, 0.407)*	—	0.329 (0.253, 0.407)*

DV, dependent variable; Ref., reference category; CI, confidence interval.

a The CI did not include a null value (*β* = 0).

## 4 Discussion

In this study, the overall magnitude of anxiety was 74.56% (95% CI = 71.78%, 77.01%), which was higher than the studies reported from Columbia at 59% (62) and Ethiopia (Gedeo Zone) at 59.1% (63) and lower than the study conducted in Nepal at 80% (22) and Afghanistan at 84.6% (23). This variation could be due to the different measurement tools used for measuring anxiety symptoms. The GAD-7 used in this study is the most sensitive tool for detecting anxiety disorder (64). It had higher sensitivity and specificity than the Hospital Anxiety and Depression Subscale (HADSS) used in a previous study conducted in Ethiopia (Gedeo Zone). A study conducted in Columbia used the Zung Anxiety Scale (ZAS), while studies conducted in Nepal and Afghanistan used the Hopkins Symptom Checklist-25 (HCL-25) to detect the symptoms of anxiety. This difference in measuring tools may have caused the variations (22, 23, 62).

Besides these, the cutoff point used for each tool may cause such variation. Furthermore, these discrepancies might result from cultural differences in different parts of the world. Another possible explanation might be the stressors experienced after displacement, such as mental health conditions that added to the burden of resettling in a new place. This process can create a considerable amount of stress for the newly displaced persons trying to restart living in new places, often resulting in anxiety (65).

In this study, we found that age, sex, occupational status, frequency of displacement, and death of a beloved one during displacement had a significant direct positive effect on anxiety, while educational status, no medically confirmed chronic illness, and social support had a direct negative effect on anxiety.

This study revealed that age had a direct positive effect on anxiety. This finding is supported by the study conducted among IDPs in Kenya (2). The plausible reason may be the variety of factors, such as aging-related changes in the brain and neurological system and a higher propensity to encounter stressful life events that can cause anxiety in IDP camps (66).

Our study findings showed that being female had a direct positive effect on anxiety. This study result was in agreement with a study conducted in Nepal (22). The possible reason could be that women may be more susceptible to life stressors than men, such as psychological and physical stress. Because of gender-based discrimination and increased vulnerability to gender-based violence, women are much more likely than men to experience stressors in their lives. These elements may have significant psychological repercussions, including elevated anxiety (67). Additionally, it might be explained by the fact that women have greater hormonal fluctuation, which makes them more vulnerable to anxiety (68).

The finding of this study showed that being displaced two times had a direct positive effect on anxiety. This finding is supported by the study conducted in Ukraine (69). This may be justified by displacement putting the IDPs, in general, in a vulnerable situation; they have a harder time finding work and cannot independently meet their fundamental necessities (70).

This study revealed that having a history of the death of a beloved one had a direct positive effect on anxiety, implying that the findings also showed that people who had lost family members through internal displacement had high levels of anxiety. This is supported by the study done in Nigeria (71). The possible reason could be that the effects of losing a loved one can be comparable to those of trauma victims who have experienced other types of trauma, including reminders of the event and intrusive negative thoughts like ideas of retaliation, and it may have a significant impact on mental health (39).

The current finding revealed that being unable to read and write and having no medically confirmed chronic illness had a direct negative effect on anxiety. They were a protective factor for anxiety, and this was an unexpected or surprising finding that those with a lower educational attainment or education had lower anxiety symptoms. This could suggest that educated displaced people might be excessively worried about finding the means to maintain themselves and sources of income in the new place.

This study indicated that having an occupation as a farmer had a direct positive effect on anxiety. Concerning occupation, an internally displaced person with an occupation as a farmer had a high level of anxiety. The possible justification might be that they were more worried about it because they had left their occupation before displacement (31).

This finding revealed that social support had a direct negative effect on anxiety. This could be elaborated with the study done in Southern Ethiopia (63). It might be because the need for social support is typically greater in internally displaced persons, and its absence during or after periods of displacement is the most reliable indicator of the detrimental effects of anxiety (72).

In the current study, the magnitude of Post-traumatic Stress Disorder using the PTSD Checklist for DSM-5 (PCL-5) measurement tool was 76.9% (95% CI: 74.2%, 79.4%). This is in line with the study conducted in Maiduguri, Nigeria, at 78% (73). This similarity could be due to study setting, sample size, and study population. However, our findings were higher than the studies conducted in Uganda at 54% (74), in Debere Berhan, Ethiopia, at 67.5% (41), and in Southern Ethiopia at 58.4% (38) and lower than a study conducted in Medellin, Colombia, at 88% (62). This discrepancy may be caused by the PTSD screening tools utilized—for instance, the Harvard Trauma Questionnaire (HTQ) was used for the study conducted in Uganda, which was a previous version of DSM-5 (PCL-5) to measure PTSD. Moreover, the discrepancies could be due to the differences in the impact of war and mass displacement in the area. Furthermore, the variations in PTSD rates reported in other research could be caused by a variety of elements, including various methodological approaches. Additionally, the duration of conflict and displacement can cause such variations.

In our study, age, sex, educational status, occupational status, frequency of displacement, and death of a beloved one had a significant positive effect on PTSD, while having no medically confirmed chronic illness and social support had a negative effect on PTSD. Anxiety also had a direct effect on PTSD. Age, sex, educational status, and death of a beloved one were significantly related to PTSD both directly and indirectly, while occupational status, frequency of displacement, no medically confirmed chronic illness, and social support were significantly related to PTSD indirectly.

This study revealed that age had a positive effect on PTSD. This finding was supported by a previous study conducted in South Sudan (75). This could be due to the older adults who were exposed to traumatic events earlier in life and had symptoms in the wake of exposure that were not identified, and it can be challenging for displaced individuals, especially those who are older, to start over from scratch after losing everything they owned (22).

Our study showed that being female had a positive effect on PTSD. This finding is supported by the study conducted in Southern Ethiopia (38). This may be because of the lower threshold for exposure to psycho-trauma than men; thus, women may have a higher risk of acquiring PTSD (62). Furthermore, PTSD was more prevalent among women IDPs, with them experiencing psycho-trauma and developing PTSD symptoms compared to males. Another reason could be women’s early recognition of psychological suffering and their ability to seek care for it (76).

The current study showed that being unable to read and write had a positive effect on PTSD. This is supported by the study conducted in Sri Lanka (77). This might be because the development of language and communication skills, the capacity to organize and retain new information, and reading comprehension are just a few of the talents that trauma can compromise (78).

This study revealed that being displaced two times had a positive effect on PTSD. This suggested that participants who had been displaced two times were more prone to suffer from PTSD than those who had only been displaced once. This finding is supported by different studies conducted in Ukraine (69). The possible justification might be that the respondents who had experienced repeated displacement were more exposed to trauma and violence than the participants who had only been displaced once. As a result, repeated exposure to trauma connected to displacement may raise the risk of developing PTSD (77).

In this study, having no medically confirmed chronic illness had an indirect negative effect on PTSD. This indicates that displaced individuals who had no medically confirmed chronic illness had less risk of PTSD compared to those who have medically confirmed chronic illness, which is protective. This could be explained by the fact that if IDPs had medically confirmed chronic illness, for example, a distressing medical diagnosis of an illness or disease, such as cardiovascular problems, cancer, or diabetes mellitus, these illnesses can increase the risk of resulting in PTSD.

This study showed that having a history of the death of a loved one had a positive indirect effect on PTSD. This indicates that participants who had a history of the death of a loved one during the displacement had greater symptoms of PTSD than those who were not. This study result was consistent with a similar study done in Nigeria (71). The plausible reason for this may be that the effects of losing a loved one can be similar to those of trauma victims who have experienced other types of trauma, including reminders of the event and intrusive negative thoughts such as ideas of retaliation, and it may have a substantial influence on mental well-being.

In this study, social support had an indirect negative effect on PTSD through anxiety. In other words, internally displaced persons with low social support experienced more PTSD than those with high social support. This may be due to the reason that a lack of social support makes people feel more alone and worthless, which can result in hopelessness, low self-esteem, and negative thoughts, which lead to PTSD, and PTSD subsequently leads to depression (29).

This finding showed that anxiety had a significant direct effect on PTSD. As the level of anxiety increases, one may have the chance to have increased PTSD symptoms. This finding was supported by other studies conducted in Ethiopia (57, 79). This could be because individuals with anxiety in the IDP camp may have excessive unwanted memories of the stressful experience which leads to PTSD. It might be caused by the way of being internally displaced from receiving support and treatment, which may cause PTSD (36).

In this study, the magnitude of depression was 79.53% (95% CI: 76.93%, 81.86%). It is consistent with the study conducted among internally displaced people in Nepal 80% (22). However, this finding was higher than the study conducted in Gedeo at 18.5% (63), Sudan at 24.3% (80), Columbia at 41% (62), and Afghanistan at 39% (81). In contrast to this, it was lower than the study conducted in Maiduguri, Nigeria, at 96.1% (73). The reason for these variations may be caused by the depression screening tools used. A study conducted in the Gedeo Zone used the HADSS, a study conducted in Sudan used DSM-IV, the Fourth Edition of the Statistical Manual for epidemiological studies, a study conducted in Columbia used the Zung Depression Scale, and a study in Afghanistan used the Hopkins Symptom Checklist-25 as screening tool used to detect the symptoms of depression, but in this study, PHQ-9 was used.

From our final SEM, having no medically confirmed chronic illness, age, sex, and anxiety were all directly and indirectly related to depression. PTSD had a direct significant relation with depression, while occupational status, frequency of displacement, death of a loved one during the displacement, and social support had a significant indirect effect on depression.

This study indicated that age had a total positive effect on depression through anxiety. The results of the study conducted in Nigeria (73) among IDPs confirm the fact that older individuals had poor mental health such as anxiety, PTSD, and depression. This result is consistent with the findings of Afghanistan (81) and from other investigations (22, 63, 81). This could be due to the fact that older persons tend to have more obligations and social stressors, especially IDPs who may experience numerous difficulties with housing, accommodations, and other aspects of social life in their new place (82).

Our study showed that being female had a positive effect on depression. The finding was supported by the study result in Nigeria (73). This could be because of factors like psychological rumination, higher levels of interpersonal neuroticism in women, and societal challenges brought on by inadequate support for them (83).

This finding showed that having a history of experiencing the death of a loved one had an indirect positive effect on depression. This implied that IDPs who had experienced the death of a loved one had higher levels of depression than those who did not. This is supported by a similar study conducted in Uganda (74).

In this finding, the indirect effect of social support through anxiety had a negative effect on depression. Internally displaced persons with low social support in the IDP camp had higher levels of depression compared to those who had high social support. This is consistent with the systematic review and meta-analysis conducted on the epidemiology of depression among internally displaced persons (29).

This revealed that anxiety had a significant positive effect on depression, which means that anxiety and depression among internally displaced persons had a positive relationship. In other words, high levels of anxiety were strongly linked to greater levels of depression. The finding is consistent with the prior study conducted in the Gaza Strip (84). The possible reason could be how people are bothered by their memories of past traumatic events and/or anxiousness by how much a person considers as intrusive memories and anxiety symptoms, thus causing their depression (85).

This study revealed that PTSD had a direct positive effect on depression, implying that IDPs who had PTSD symptoms had an increased level of depression. This finding is consistent with a previous study conducted in Nigeria (73). The possible justification for this may be that individuals after trauma and more trauma may have experienced or had a greater chance of developing anxiety leading to PTSD (45). Some people can feel depressed after traumatic events, particularly during displacement, whether they physically experience them or only hear about them, which leads to a higher level of depression (44). Furthermore, a risk factor for mental illness is exposure to trauma, in addition to the genetic, biochemical, and psychological predisposition to mental illness (81).

The strongest aspect of the study was the simultaneous assessment of the direct and indirect impacts of multiple independent variables on the dependent variables of post-traumatic stress disorder, depression, and anxiety. However, there were limitations to the study. Recall bias may have occurred, and the study’s generalizability to all internally displaced people may have been impacted by the fact that it only included adult internally displaced people.

Studies outside of internally displaced persons are recommended. Medical practitioners must understand the connection between post-traumatic stress disorder, depression, and anxiety. Internally displaced persons should seek medical attention if they experience signs of post-traumatic stress disorder, depression, or anxiety. Research on a mixed approach to internally displaced people is advised for researchers.

### 4.1 Strength and limitation

This study assessed the magnitude and determinants of mental health disorders—anxiety, post-traumatic stress disorder, and depression—among the samples of internally displaced persons in the Metekel Zone. In this study, the mental health disorders—anxiety, PTSD, and depression—were assessed using standardized tools that were validated in both developed and developing countries. Moreover, the current study was employed using structural equation modeling which enables the assessment of direct and indirect effects on multiple outcome variables simultaneously. However, this study had its limitations. Only internally displaced persons found in IDP were included in this study, which may affect the generalizability for all internally displaced persons. The cross-section nature of the study makes it challenging to determine the connections between anxiety, PTSD, and depression.

## 5 Conclusion

This study was conducted to assess the magnitude and determinants of mental health disorders—anxiety, post-traumatic stress disorder, and depression—among the samples of internally displaced persons in the Metekel Zone. The magnitude of anxiety, PTSD, and depression was found to be high. Older-aged IDPs, female IDPs, and those who were not supported by friends or the government were found to be most at risk.

Age, sex, educational status, occupational status, medically confirmed chronic illness, frequency of displacement, and social support had a direct relation with anxiety, while age, sex, educational status, and death of a beloved one were significantly related to PTSD both directly and indirectly, and occupational status, frequency of displacement, no medically confirmed chronic illness, and social support were significantly related to PTSD indirectly. Anxiety was directly related to PTSD.

Having no medically confirmed chronic illness, age, sex, and anxiety had a significant relation with depression both directly and indirectly. PTSD had a direct significant relation with depression, whereas occupational status, frequency of displacement, death of a beloved one, and social support had a significant indirect effect on depression.

### 5.1 Recommendation

Strengthening the promotion of mental health practices for internally displaced people (IDPs) resulting from conflict is necessary, particularly for those who have a history of losing loved ones, have little social support, and are female. It would be better to plan psychological interventions for the IDPs in order to improve their living conditions and help them deal with the consequences of psycho-trauma. It is preferable to set up a mental health clinic in the camp to help internally displaced people who are suffering from depression, PTSD, and anxiety.

## Data Availability

The raw data supporting the conclusions of this article will be made available by the authors, without undue reservation.
